# Supplementary material for: Comparisons of Estimated Intakes and Plasma Concentrations of Selected Fatty Acids in Pregnancy
Source: Nutrients. 2019 Mar 6;11(3):568. doi: 10.3390/nu11030568 (PMC6470916; doi:10.3390/nu11030568)

Table S1. Difference in **biomarker** concentration (%TPFA) per 1 percentage increase in relative fatty acid intake and correlations between biomarker concentration and intake from Spearman statistics unless indicated otherwise (n=255).

	**Regression coefficients (95% CI) from linear regressions**		**Correlation coefficients**
	Non-adjusted	Adjusted for age, parity, BMI, smoking and total energy intake	
**EPA**	**0.44 (0.20; 0.69)** % per 1% relative increase/day **	**0.43 (0.17; 0.68)** % per 1% relative increase/day*	**0.24** ***
**DHA**	**0.52 (0.25; 0.80)** % per 1% relative increase/day **	**0.44 (0.15; 0.73)** % per 1 % relative increase/day *	**0.21** **
**EPA+DHA**	**0.51** % **(0.26; 0.77)** % per 1% relative increase/day **	**0.45 (0.19; 0.72)** % per 1 % relative increase/day *	**0.23** **
**ALA**	0.02 (-0.02; 0.06) % per 1% relative increase/day	0.03 (-0.01; 0.06) % per 1 % relative increase/day	0.06[a]
**LA**	0.10 (-0.06; 0.27) % per 1% relative increase/day	0.06 (-0.11; 0.23) % per 1 % relative increase/day	0.08
**AA**	0.85 (-1.97; 2.66) % per 1% relative increase/day	1.26 (-1,84; 3.35) % per 1 % relative increase/day	0.06[a]

*BOLD = P <0.05, *= P <0.01, **= P <0.001, ***= P <0.0001*

[a]: *Pearson correlation*

AA: arachidonic acid; ALA: α-linolenic acid; BMI: body mass index; DHA: docohexaenoic acid; EPA: eicosapentaenoic acid; LA: linoleic acid; %TPFA: percentage of total plasma fatty acids

Table S2a. Difference in **biomarker** concentration (%TPFA) per increase in daily intake and correlations between biomarker concentration and intake from Spearman statistics unless indicated otherwise. Stratified to samples stored at -196 ˚C since 2013 (n=203)

	**Regression coefficients from linear regressions (95% CI)**		**Correlation coefficients**
	Non-adjusted	Adjusted for age, parity, BMI, smoking and total energy intake	
**EPA**	**0.05 (0.01; 0.08)** % per 100 mg/day *	**0.05 (0.01; 0.08)** % per 100 mg/day *	**0.22** *
**DHA**	**0.04 (0.00; 0.07)** % per 100 mg/day	**0.04 (0.00; 0.07)** % per 100 mg/day	**0.15**
**EPA+DHA**	**0.04 (0.01; 0.08)** % per 100 mg/day *	**0.04 (0.01; 0.07)** % per 100 mg/day *	**0.18** *
**ALA**	**0.03 (0.00; 0.05)** % per 1 g/day	**0.07 (0.03; 0.11)** % per 1 g/day **	**0.15**[a]
**LA**	0.06 (-0.06; 0.19) % per 1 g/day	0.04 (-0.20; 0.27) % per 1 g/day	0.06
**AA**	<0.001 (-0.03; 0.03) % per 10 mg/day	0.01 (-0.02; 0.04) % per 10 mg/day	<0.01[a]

Model 2: Adjusted for age, parity, BMI, smoking and total energy intake
*BOLD = P <0.05, *= P <0.01, **= P <0.001, ***= P <0.0001*

[a]: *Pearson correlation*

AA: arachidonic acid; ALA: α-linolenic acid; BMI: body mass index; DHA: docohexaenoic acid; EPA: eicosapentaenoic acid; LA: linoleic acid; %TPFA: percentage of total plasma fatty acids

Table S2b. Plasma sample storage temperatures (n=252)

Storage temperature	n	%
**-196 ˚C**	203	80.6
**-80 ˚C**	40	15.9
**-20 ˚C**	9	3.6

Figure S1. Quartile mean intake of EPA+ DHA (g/day) and the corresponding plasma concentration of EPA+DHA (% TFA) (n=255).

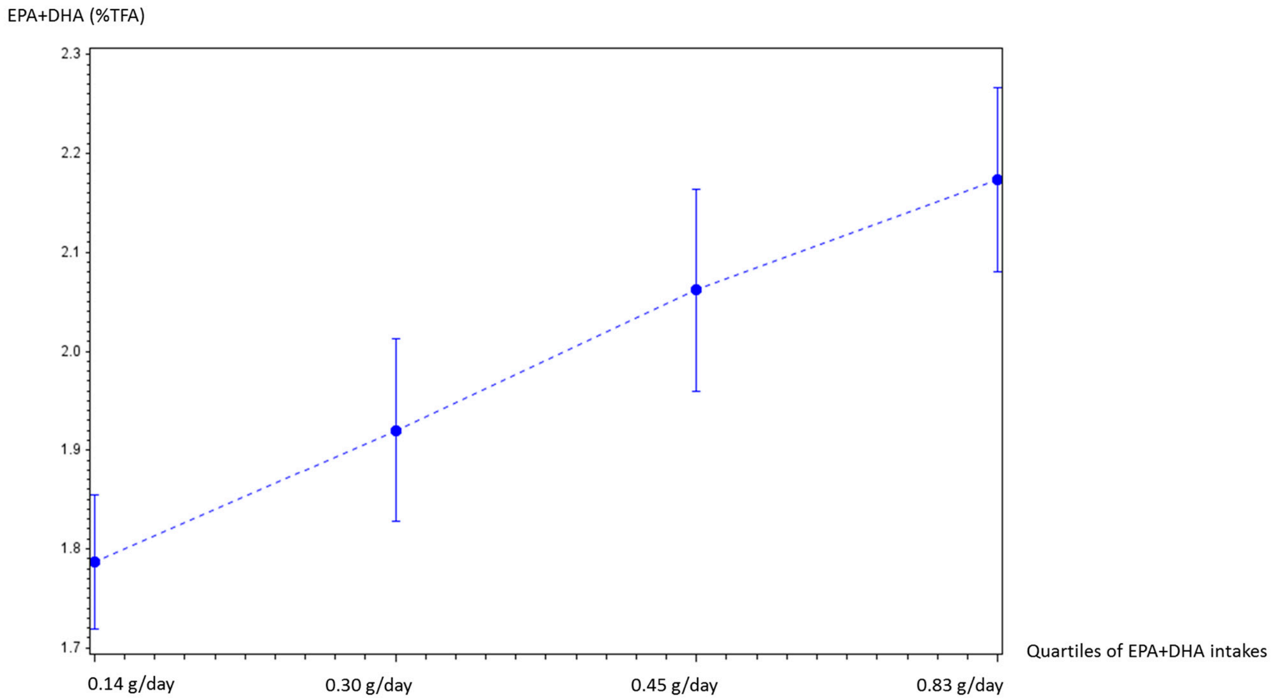

Supplement: Supplementary file 1 [file nutrients-11-00568-s001.pdf]
